# Ecological study on household air pollution exposure and prevalent chronic disease in the elderly

**DOI:** 10.1038/s41598-023-39059-9

**Published:** 2023-07-20

**Authors:** Samaneh Dehghani, Somayeh Yousefi, Vahide Oskoei, Moslem Tazik, Mohammad Sanyar Moradi, Mahmood Shaabani, Mohebat Vali

**Affiliations:** 1 Department of Environmental Health Engineering, School of Public Health, Tehran University of Medical Sciences, Tehran, Iran; 2 Student’s Scientific Research Center, Tehran University of Medical Sciences, Tehran, Iran; 3 School of Life and Environmental Science, Deakin University, Geelong, Australia; 4 Department of Occupational Health and Safety Engineering, School of Health, Shiraz University of Medical Sciences, Shiraz, Iran; 5 Education (and Training) Office of Hendijan, Hendijan, Khuzestan Iran; 6 Department of Epidemiology, School of Health, Shiraz University of Medical Sciences, Shiraz, Iran; 7 Student Research Committee, Shiraz University of Medical Sciences, Shiraz, Iran

**Keywords:** Environmental sciences, Risk factors

## Abstract

Older people spend most of their time indoors. Limited evidence demonstrates that exposure to indoor air pollutants might be related to chronic complications. This study aimed to estimate the correlation between household air pollution (HAP)'s long-term exposure and the prevalence of elevated hypertension, diabetes mellitus (DM), obesity, and low-density lipoprotein (LDL) cholesterol. From the Global Burden disease dataset, we extracted HAP, hypertension, DM, body mass index, and LDL cholesterol data from Iran from 1990 to 2019 to males and females in people over 50 years. We present APC and AAPC and their confidence intervals using Joinpoint Software statistical software. R software examined the correlation between HAP and hypertension, DM2, Obesity, and high LDL cholesterol. Our finding showed a significant and positive correlation between HAP exposure and prevalence of high low-density lipoprotein cholesterol (p ≤ 0.001, r = 0.70), high systolic blood pressure (p ≤ 0.001, r = 0.63), and high body mass index (p ≤ 0.001, r = 0.57), and DM2 (p ≤ 0.001, r = 0.38). The analysis results also illustrated a positive correlation between indoor air pollution and smoking (p ≤ 0.001, r = 0.92). HAP exposure might be a risk factor for elevated blood pressure, DM, obesity, and LDL cholesterol and, consequently, more serious health problems. According to our results, smoking is one of the sources of HAP. However, ecological studies cannot fully support causal relationships, and this article deals only with Iran. Our findings should be corroborated in personal exposure and biomonitoring approach studies.

## Introduction

Household air pollution (HAP) is a main cause of death, with 3.8 million deaths annually^1^. Concentrations of HAP by significant human health effects^2^ in places such as the home, workplace, restaurants, and schools can be considered the most appropriate exposure measure in studies^3,4^. 95% of older people's time is spent at home and indoors. Inadequate ventilation is a compelling dilemma in nursing homes^5^. In addition to the kinds of used fuel for cooking and the source of indoor heating energy, smoking, passive exposure to cigarette smoke and tobacco^6^, and outdoor air pollution can also be considered sources of indoor air pollution^7^. Some indoor air pollutants include radon, NO_x_, SO_2_, O_3_ (VOCs), PM, CO, and microorganisms^7^. Generally, air pollution can be inorganic, organic, biological, and radioactive^8,9^. Some pollutants, such as NO_x_, SO_2_, O_3_, and PM, are common indoors and outdoors. The concentrations of outdoor and indoor air pollutants are related to individual exposure to diverse environments^10^.

Recent studies showed a strong correlation between indoor air quality and smoking, cooking, and using fireplaces^11^. Cooking activities can increase the PM by about 1.5 to 27 times. In addition, there is some difference between the structure, chemical, and physical properties of indoor PM2.5 and outdoor PM2.5 and different health effects^12^. Due to the accumulation of pollutants, the absence of proper ventilation can severely affect residents' health^12^. Some acute effects include eyes, noses, allergies, skin diseases, throat irritation, etc.^13^. Current studies have presented evidence of the association between increased death and long-term exposure to low concentrations of PM2.5 (< 30 μg/m^3^)^14–16^.

Because most patients are DM2, associations are shown mostly the effect of type 2. A Correlation between PM exposure and diabetes was shown in some studies^17^. Evidence of air pollution's impact on diabetes is increasing, but most inquiries have been reported in high-income countries as the air pollution rate is high^18,19^. Short-term and long-term exposure to air pollution through the mechanism of inflammation, oxidative stress, and arterial regeneration can be associated with diastolic blood pressure (DBP) and systolic blood pressure (SBP)^20,21^. There is some report of a correlation between rising body mass index (BMI) and levels of PM_2.5_, PM_10_, NO_2_, NO_x_, and PM_2.5_ in the past literature^22–24^.

Forecasting Iran's aging population will be 10.5% in 2025 and 21.7% in 2050, the oldest people in the region by 2050^25^. Also, due to the high cost and the need for labor in indoor sampling, indoor air quality studies usually include information on a few homes that may not be well indicative of the exposure^7^. Therefore, considering the significance of indoor air pollution in the prevalence of adverse consequences of exposure, especially for the elderly, the present study intended to estimate the association between HAP and the majority of diabetes, hypertension, LDL cholesterol, and obesity in the Iranian population over 50 years, in 1990 to 2019 as an ecological study.

## Materials and methods

### Collecting data

Data were gathered separately from 1990 to 2019 based on sex and age groups 50–69 years and over 70 years, correlated to the prevalence of DM2, hypertension, and high BMI (body mass index). In addition, the death of risk factors, including tobacco use, smoking, drug use and alcohol use, was collected.

The data for systolic blood pressure (SBP) were measured in mmHg of 110 to 115 mmHg was used for TMREL SBP. For adults aged 20 and above, body mass index (BMI) is defined as greater than 20–25 kg/m^2^ of height. Diabetes mellitus is an example of a metabolic disease characterized by high glucose levels (hyperglycemia). The exposure to indoor air pollution was categorized based on the use of solid fuel for cooking, also known as HAP^26^. All data is collected from the official websites: http://www.healthdata.org/^26^.

According to the GBD 2019 study, this website presents injuries and risk factors from 1990 to 2019 in 204 countries. Finally, overall, 369 causes of illness and injury were systematically analyzed in this website. Details of the methodology and the main changes incorporated are described elsewhere^27^ and part of the data was also collected from the STEPS data based on the website https://vizit.tums.ac.ir/panel/steps-2020/en/main.html#/map.

### Statistical analysis

After descriptive analysis, the Joinpoint regression model was used to show the trend status of the prevalence of DM2, in the Joinpoint software version 4.9.0.0. In addition, Annual Percent Change (APC) and Average Annual Percent Change (AAPC) were reported separately for men and women with their confidence intervals (CI). The significance level in all analyzes was 0.05.Another analyzes were performed using R software (version 3.5.0) using the Spearman correlation test. Interpretation of correlation relationship intensity was based on r = 0.8–1; very strong. r = 0.6–0.08; strong, r = 0.4–0.6; moderate, and r = 0.2–0.4; low or weak correlation relationship^28^. The maps were drawn using Datawrapper.

## Results

### Descriptive results of prevalence data

**Figure 1:**
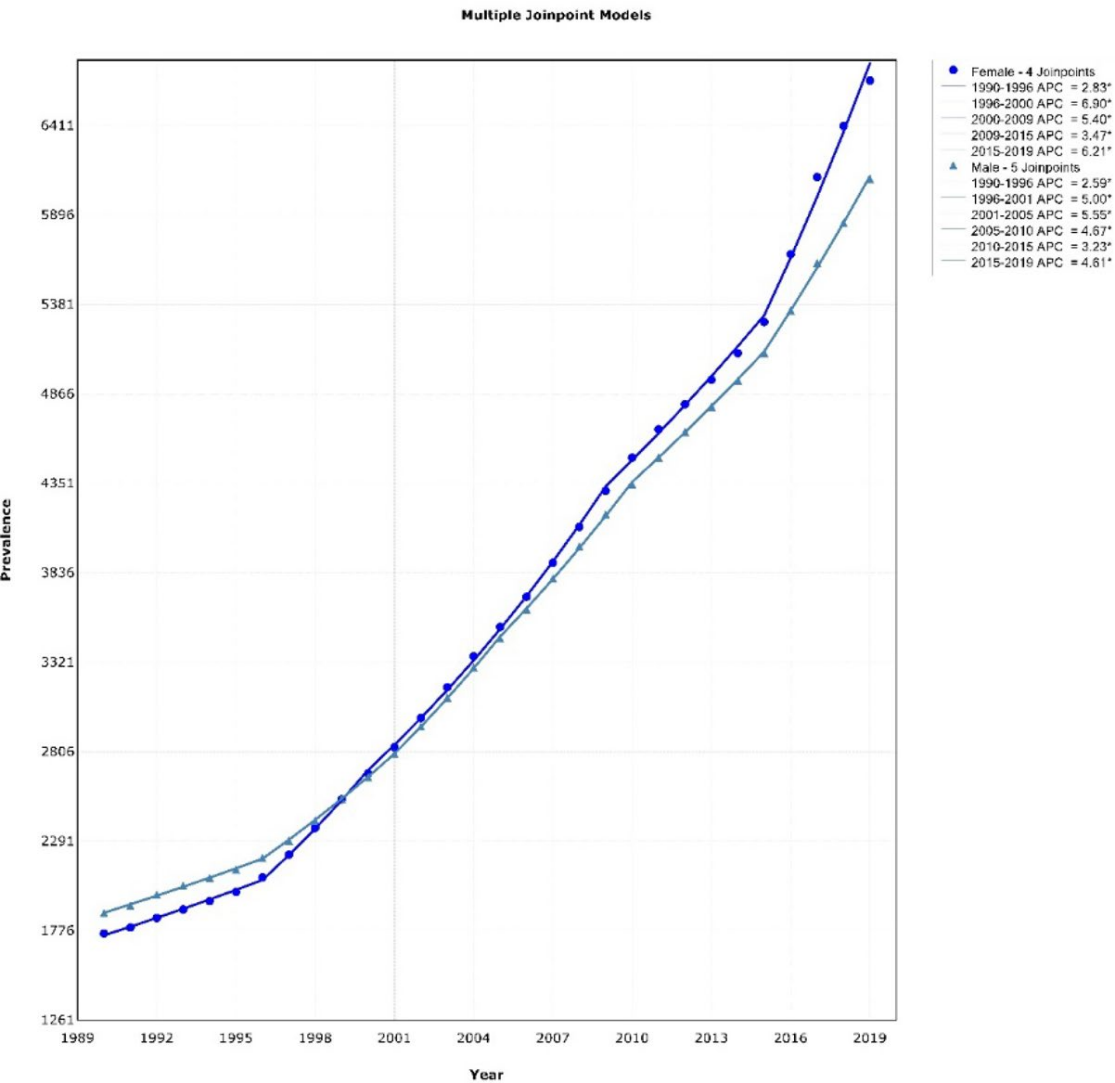
Prevalence of diabetes mellitus in Iran in people over 50 years, 1990–2019.

**Figure 2:**
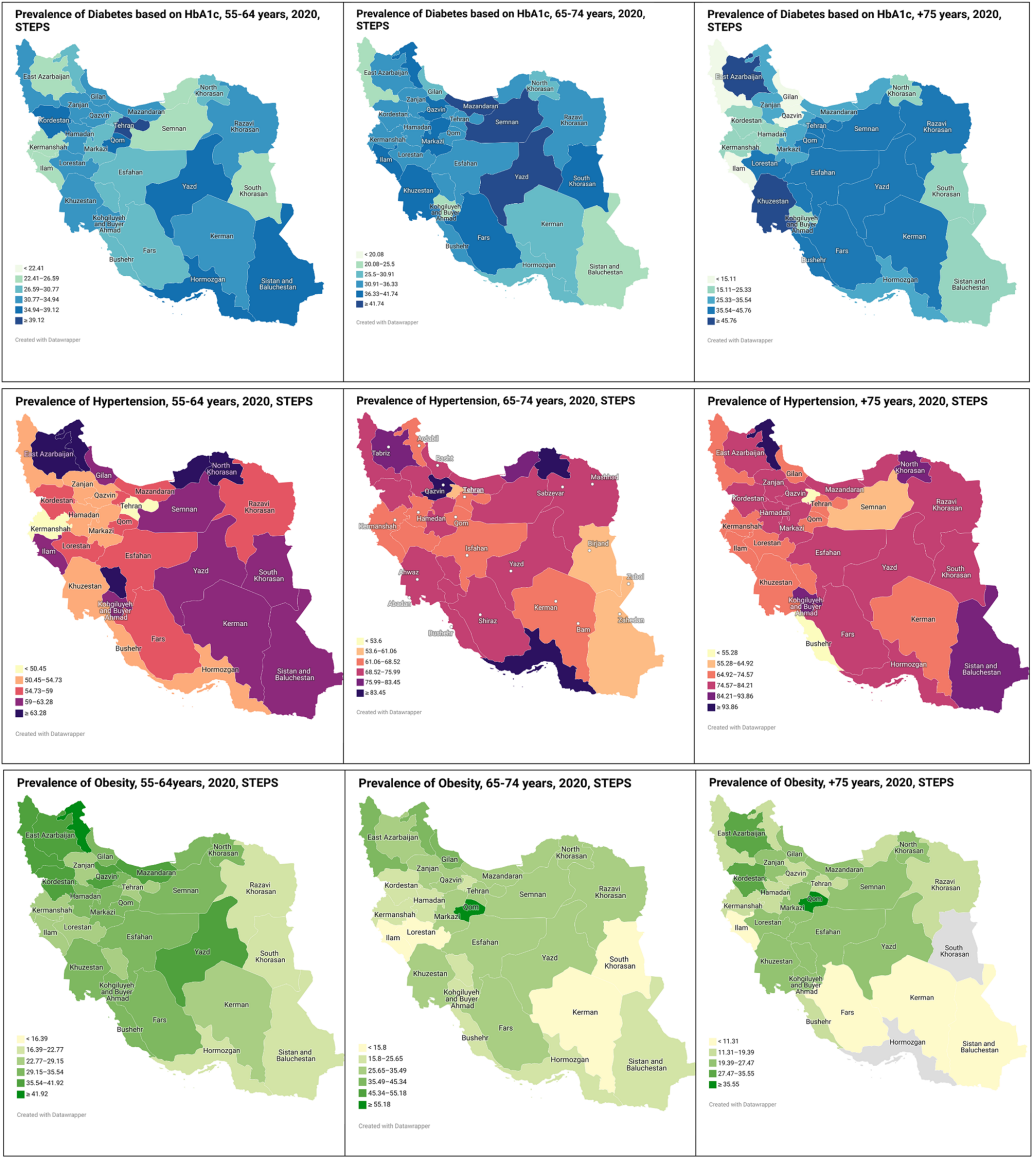
Distribution of Diabetes, Hypertension, and obesity in IRAN for 55–64, 65–74 and ≥ 75 years in 2020.

Descriptive results of the DM2 prevalence from 1990 to 2019 by gender in people over 50 years were shown in Fig. 1. These results indicated that the prevalence of DM2 in males had five joinpoints, and its general trend was upward [AAPC = 4.78% (CI 4.61–4.95)] and had four joinpoints in females, and the general trend was upward. [AAPC = 4.16% (CI 4.07–4.24)]. Descriptive results of DM2 prevalence also showed a strong upward trend in DM2 prevalence in women from 2015 to 2019 (APC = 6.21%). The descriptive results of the distribution of DM2 prevalence based on the HbA1c test, hypertension and obesity, by Iranian people 55–64, 65–74 and ≥ 75 years in 2020 (per 100,000) were shown in Fig. 2. For the age group of 55–64 years, the highest prevalence of DM2, hypertension and obesity were in Tehran, North Khorasan and Ardabil provinces, respectively. For the age group of 65–74 years, it was in Semnan, Hormozgan and Qom, respectively. For the age group over 75 years, it was in Khuzestan, Ardabil and Qom, respectively.

### Indoor air pollution and prevalence of DM2, high SBP, obesity, and high LDL cholesterol

**Figure 3:**
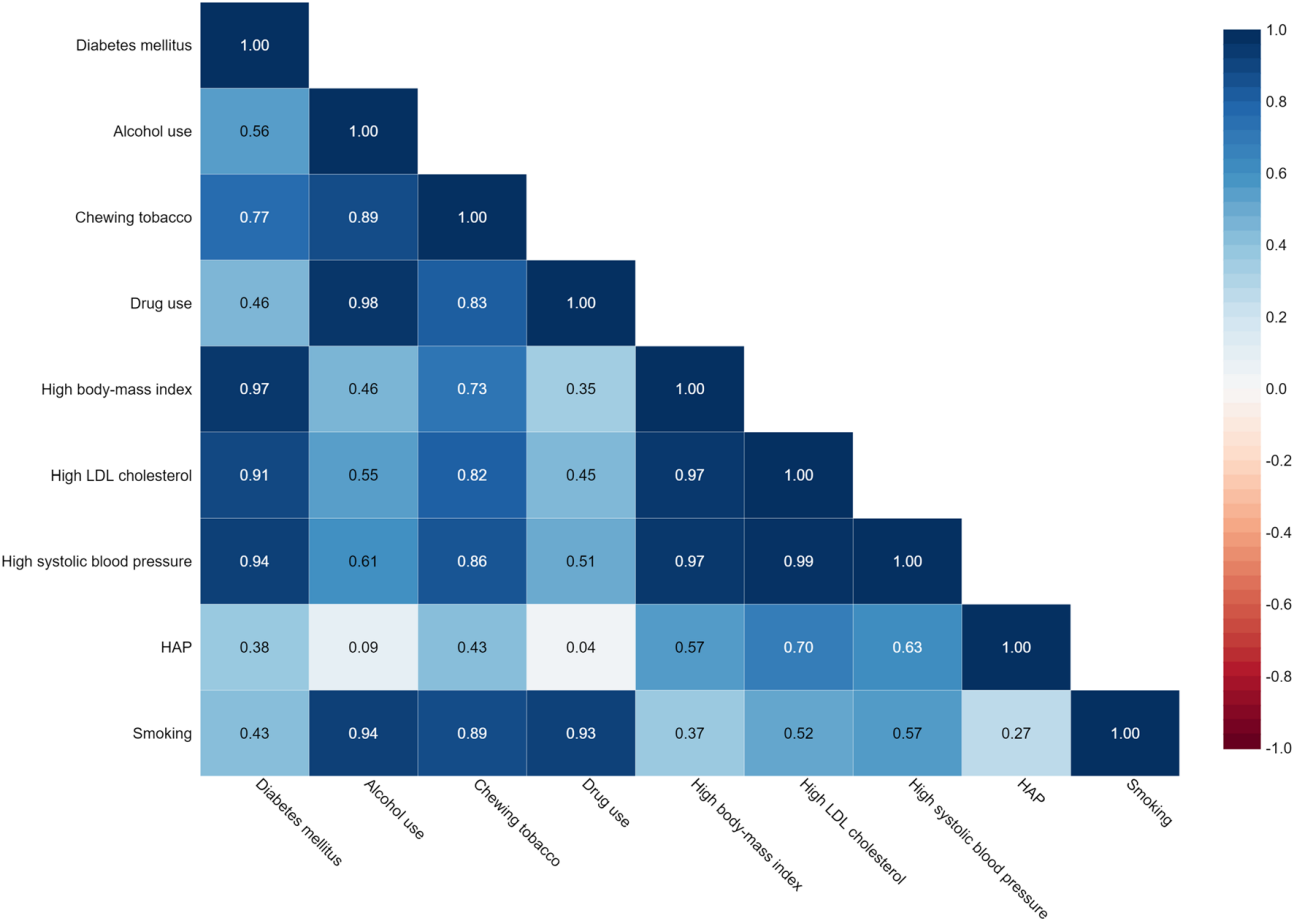
Correlation of exposure to HAP with Diabetes, Hypertension, High LDL Cholesterol, and Obesity in IRAN.

The related results between HAP exposure and the prevalence of DM2, high SBP, obesity, and high LDL cholesterol in the population over 50 years are shown in Fig. 3. A significant relationship positive correlation was discovered between HAP and DM2 prevalence (r = 0.38, p ≤ 0.001), high SBP (r = 0.63, p ≤ 0.001), high LDL cholesterol (r = 0.70, p ≤ 0.001) and high BMI (r = 0.57, p ≤ 0.001).

### Correlation between the traditional risk factors and prevalence of DM2, high SBP, obesity, and high LDL cholesterol

The correlation between the prevalence of DM2, high SBP, obesity, and high LDL cholesterol in over 50 years population and the traditional risk factors for mentioned diseases, including smoking, drug use, alcohol consumption, and tobacco use was shown in Fig. 3. Correlation analysis results, significant relationship, and positive correlation were observed between smoking and prevalence of high SBP (r = 0.57, p ≤ 0.001), high LDL cholesterol levels (r = 0.52, p ≤ 0.001), high BMI (r = 0.37, p ≤ 0.001) and DM2 (r = 0.43, p ≤ 0.001). Correlation results showed a significant relationship positive correlation between tobacco use and prevalence of high SBP (r = 0.86, p ≤ 0.001), high levels of LDL cholesterol (r = 0.82, p ≤ 0.001), high BMI (r = 0.73, p ≤ 0.001), and DM2 (r = 0.77, p ≤ 0.001). Correlation analysis also showed a significant relationship positive correlation between alcohol consumption and prevalence of high SBP (r = 0.61, p ≤ 0.001), high levels of LDL cholesterol (r = 0.55, p ≤ 0.001), high BMI (r = 0.46, p ≤ 0.001), and DM2 (r = 0.56, p ≤ 0.001). In addition, Significant relationship positive correlation between drug use and high SBP prevalence (r = 0.51, p ≤ 0.001), high levels of LDL cholesterol (r = 0.45, p ≤ 0.001), high BMI (r = 0.97, p ≤ 0.001), and DM2 (r = 0.46, p ≤ 0.001) were the results of the correlation.

### Relation between HAP and smoking, tobacco, and drug use

The correlation between HAP and smoking, tobacco, and drugs as risk factors for the mentioned disease are shown in Fig. 3. The results of Spearman correlation analysis showed a significant positive relationship between HAP and smoking (r = 0.92, p-Value ≤ 0.001). Therefore, according to the results of this study, smoking is one of the sources of HAP, an essential risk factor for DM2, high SBP, high levels of LDL cholesterol, and high BMI.

## Discussion

About 3 billion people worldwide (especially in developing countries) have used fossil fuels for cooking and heating^29^. The association of HAP exposure with various diseases and premature death has been reported in previous studies^30–32^. It is estimated that approximately 2.8 million premature deaths worldwide result from exposure to HAP^33^. In summary, this study achieved two goals: (1) evaluating the situation of HAP exposure in Iran from 2000–2019, and (2) examining the correlation between HAP exposure and the prevalence of DM2, hypertension, obesity, and high LDL cholesterol in over 50 years old in Iran. The relationship between the mentioned disease's prevalence and HAP exposure was investigated with risk factors such as smoking.

Incomplete combustion of solid fuels and tobacco use produces various pollutants such as CO, SO2, PM, carbon black (BC), toxic metals, and PAHs^34–36^, of which PM and PAH are important productions^37,38^. High emissions of indoor air pollutants may also be correlated with ambient air pollution^39^. Factors that are influenced by lifestyle in HAP exposure are the interior design of the kitchen, stove design, utilizing ventilation, cooking frequency, and passive smoking^40^. Rapid urbanization and industrialization have changed the structure of household energy consumption for cooking and heating^41^. According to Lee et al., living rooms and bedrooms must be separated from the kitchen. Living areas must be adequately ventilated to reduce exposure to air pollution^40^. Although in controlling air pollution, some ignore residential combustion compared to other sources of pollution, such as power plants and automobile emissions, HAP and its effects have raised growing concerns^42^.

Our study showed a significant relationship and positive correlation between HAP exposure and high SBP prevalence in the population aged 50 years and older in Iran. Several systematic studies have been conducted on HAP effects on health^31,43,44^. Exposure to indoor air pollutants can lead to absorption into the human body and accumulation in hair and lipid tissues^45,46^. PM inhalations have been outlined as a pathway for numerous metals to enter the body of humans^47^. Therefore, examining whether the metals uptake due to HAP exposure is related to blood pressure is necessary. Hypertension is considered the leading risk factor for heart disease globally, affecting more than one billion people globally, and 9.4 million new cases are recorded annually^48^. Besides some hypertension risk factors, including physical activity, smoking, and diet, environmental factors, notably air pollution, have been considered worldwide^49,50^. HAP Exposure caused by household solid fuel combustion is related to an increased risk of hypertension^51^. It increases the likelihood of detecting high BP in China^52^. Using biomass instead of liquefied petroleum gas increases particulate matter and the prevalence of hypertension^53^. HAP levels vary household by household and are challenging to monitor. Some inquiries reported the relationship between ambient air pollution and the risk of hypertension, but less research has been conducted about the association between HAP and the risk of hypertension. The results of our study illustrated a significant relationship and positive correlation between HAP exposure and DM2 prevalence in the population aged 50 years and older in Iran. DM2 is a conventional disease globally, and its prevalence has been rising in recent years^54^. Although obesity plays an important role in increasing DM2 prevalence, air pollution is also considered a potential risk factor for DM2^55,56^. However, the effect of air pollution is not clearly described. The association between chronic exposure to air pollution, impaired glucose metabolism, development of insulin resistance, and the risk of DM2 has been assessed in several inquiries. However, this association is still unclear due to conflicting results^57–60^. Possible mechanisms include oxidative stress and persistent inflammation, with impaired insulin signaling, eventually leading to diabetes mellitus^61^.

This study's correlation analysis shows a high BMI correlation and HAP exposure. Obesity is a chronic non-communicable disease associated with the risk of other diseases^62^. It has been observed that environmental stressors such as endocrine-disrupting chemicals can be responsible for obesity^63,64^. Air pollution is a risk factor for obesity caused by environmental factors^65,66^. Some animal studies showed air pollution could alter the body's metabolism and contribute to weight gain^67,68^. In most of these reports, increasing BMI is associated with increased levels of NO_2_, NOx, PM2.5, PM10, and PM coarse^23,69,70^. Yang et al. conducted a study on people the age of 61 years old on average to examine the association between BMI and PM_2.5_, PM_10_, O_3_, CO, NO_2_, and SO_2_ in 125 Chinese cities. PM2.5 concentration for groups with BMI < 18.5, BMI = 18.5–23.9, BMI = 24–27.9, and BMI > 28 was reported 34.68 $\mu$/m^2^, 36.12 $\mu$/m^2^, 39.18 $\mu$/m^2^, 41.07 $\mu$/m^2^ respectively^71^. Also, Li et al. illustrated a positive impact of exposure to PM2.5 on BMI^72^.

The results of our study determined the relationship between HAP exposure and high LDL cholesterol in people over 50 years old in Iran. In this regard, Yang et al. (2018) study reported the association between PM_2.5_ and NO_2_ exposure and hypercholesterolemia by examining air pollution's effect on fat levels and dyslipidemias. The results of Yang et al.'s (2018) research also illustrated that the association between air pollutants and dyslipidemias was more significant in participants who were overweight or obese^73^. Studies in rodents have also shown that exposure to PM2.5 comprises an inflammatory response in visceral adipose tissue^74,75^ or epidermal adipose tissue^76^.

This study demonstrated a significant relationship between smoking and HAP, although the results indicate a weak correlation between smoking and HAP. In our study, smoking also showed a moderate correlation with DM2 prevalence, high systolic BP, high LDL cholesterol, and obesity. Smoking is a severe concern for public health globally. Many healthcare planners try to design and implement appropriate strategies to decrease smoking as the most critical factor related to mortality^77,78^. Smoking prevalence is relatively high in Iran^79^. Literature also reported smoking had been considered an important source of HAP^7,80^. Our study results are adopted with the previous research on smoking and even exposure to secondhand smoke (a risk factor for hypertension)^21^, diabetes^81^, obesity^82^, and high LDL cholesterol^83^.

### Strengths and limitations

A global study of disease burden has estimated that HAP is liable for more than 3 million premature deaths worldwide and is the fourth most common risk factor for all deaths^32^. This research analyzed the relationship between exposure to HAP and common chronic diseases, especially in the elderly. In addition, we showed the relationship between exposure to HAP and traditional risk factors for the prevalence of these diseases. Previous studies have focused on the relationship between people's health and occupational and environmental exposures. In many cases, measuring an individual's long-term exposure to air pollutants requires a lot of time and money. We were able to solve this problem mainly by using an ecological study^84^. However, any research has its limitations. This study was done as an ecological study, and in fact, the results of our study were from a data set^85^. Generalizing the results to all people will lead us to ecological fallacies. They are considering that ambient air pollution and the cumulative impacts of air pollutants can also potentially impact the prevalence of these diseases. Various factors such as fuel and stove types, burning frequency, people characteristics, ventilation conditions, the structure of the room, different seasons, fuel/energy types, environmental and meteorological conditions, and other sources of pollution as critical factors can affect indoor air pollution^86^. However, limited information has been reported on these influential factors. Identifying key factors and perceptions of how indoor air quality changes is essential to designing an efficient solution to boost air quality.

### Suggestion

Reducing individual exposure to indoor air pollutants, especially in vulnerable populations including diabetics, people with high LDL cholesterol, high BP, and high BMI, can be done through simple measures, such as the installation of ventilation systems with filtration for houses in polluted areas, especially for people at risk, proper use of primary or secondary contraceptives to counteract the potential effects of HAP exposure^87^. The preferred method for assessing personal exposure to air pollutants is portable personal samplers with a high ability to estimate daily inhalation exposure. In addition to CO, SO_2_, and PM, exposure assessment of most air pollutants is necessary to arrange a more reliable interpretation of the health effects of exposed individuals. Investigating the source of HAP with different resource allocation methods for various pollutants can also be considered in future studies. Assessing the health consequences of HAP exposure according to the investigation of particular biomarkers and epidemiological inquiries is essential. These studies increase our knowledge of the detrimental effects of HAP and assist in assuring the public and policymakers of more severe proceedings for this issue.

## Conclusion

This inquiry is the first ecological study on exposure to indoor air pollution and the prevalence of chronic diseases in the age group over 50. This study illustrated a positive relationship between HAP exposure and the prevalence of DM2, high SBP, obesity, and high LDL cholesterol in the population over 50 years in Iran. In addition, smoking was shown to be a source of HAP and a risk factor for the diseases listed. This study was ecological and focused only on the elderly in Iran. Causal relationships cannot be fully supported through ecological studies. Fuel is one of the most important factors effective in HAP and exposure. Moreover, ventilation conditions significantly affect air quality. This issue is expected to be explored in future studies. To investigate the health effects of indoor air pollution, we use vulnerable older populations because they spend more of their time at home than others; the need for future studies with personal sampling is significantly felt. Health professionals play an essential part in supporting educational and policy initiatives as well as advising their patients.

## Data Availability

All used raw data in this study are available at http://www.healthdata.org/. The datasets analyzed during the current study are available from the corresponding author upon reasonable request.
